# Impact of self-reported Gastroesophageal reflux disease in subjects from COPDGene cohort

**DOI:** 10.1186/1465-9921-15-62

**Published:** 2014-06-03

**Authors:** Carlos H Martinez, Yuka Okajima, Susan Murray, George R Washko, Fernando J Martinez, Edwin K Silverman, Jin Hwa Lee, Elizabeth A Regan, James D Crapo, Jeffrey L Curtis, Hiroto Hatabu, MeiLan K Han

**Affiliations:** 1 Division of Pulmonary and Critical Care Medicine, University of Michigan Medical Center, Medical Center Drive, 3916 Taubman Center, Box 0360, 1500 E, Ann Arbor, MI 48109-0360, USA; 2 Department of Radiology, St. Luke’s International Hospital, Tokyo, Japan; 3 Department of Biostatistics, University of Michigan School of Public Health, Ann Arbor, MI, USA; 4 Pulmonary and Critical Care Division, Brigham and Women’s Hospital, Boston, MA, USA; 5 Channing Division of Network Medicine, Pulmonary and Critical Care Division, Brigham and Women’s Hospital, Boston, MA, USA; 6 Department of Internal Medicine, School of Medicine, Ewha Womans University, Seoul, South Korea; 7 Department of Medicine, National Jewish Health, Denver, CO, USA; 8 Medicine Service, VA Ann Arbor Healthcare System, Ann Arbor, MI, USA; 9 Department of Radiology, Brigham and Women’s Hospital, Boston, MA, USA

**Keywords:** COPD, Gastroesophageal reflux, Comorbidity, Exacerbations, Quality-of-life, Chronic bronchitis

## Abstract

**Background:**

The coexistence of gastroesophageal reflux disease (GERD) and COPD has been recognized, but there has been no comprehensive evaluation of the impact of GERD on COPD-related health status and patient-centered outcomes.

**Methods:**

Cross-sectional and longitudinal study of 4,483 participants in the COPDGene cohort who met GOLD criteria for COPD. Physician-diagnosed GERD was ascertained by questionnaire. Clinical features, spirometry and imaging were compared between COPD subjects without versus with GERD. We evaluated the relationship between GERD and symptoms, exacerbations and markers of microaspiration in univariate and multivariate models. Associations were additionally tested for the confounding effect of covariates associated with a diagnosis of GERD and the use of proton-pump inhibitor medications (PPIs). To determine whether GERD is simply a marker for the presence of other conditions independently associated with worse COPD outcomes, we also tested models incorporating a GERD propensity score.

**Results:**

GERD was reported by 29% of subjects with female predominance. Subjects with GERD were more likely to have chronic bronchitis symptoms, higher prevalence of prior cardiovascular events (combined myocardial infarction, coronary artery disease and stroke 21.3% vs. 13.4.0%, p < 0.0001). Subjects with GERD also had more severe dyspnea (MMRC score 2.2 vs. 1.8, p < 0.0001), and poorer quality of life (QOL) scores (St. George’s Respiratory Questionnaire (SGRQ) total score 41.8 vs. 34.9, p < 0.0001; SF36 Physical Component Score 38.2 vs. 41.4, p < 0.0001). In multivariate models, a significant relationship was detected between GERD and SGRQ (3.4 points difference, p < 0.001) and frequent exacerbations at baseline (≥2 exacerbation per annum at inclusion OR 1.40, p = 0.006). During a mean follow-up time of two years, GERD was also associated with frequent (≥2/year exacerbations OR 1.40, p = 0.006), even in models in which PPIs, GERD-PPI interactions and a GERD propensity score were included. PPI use was associated with frequent exacerbator phenotype, but did not meaningfully influence the GERD-exacerbation association.

**Conclusions:**

In COPD the presence of physician-diagnosed GERD is associated with increased symptoms, poorer QOL and increased frequency of exacerbations at baseline and during follow-up. These associations are maintained after controlling for PPI use. The PPI-exacerbations association could result from confounding-by-indication.

## Background

The association between gastroesophageal reflux (GERD) and chronic obstructive pulmonary disease (COPD) has been previously investigated [1]. Cross-sectional studies with limited sample size have reported, with some exeptions, that esophageal disease-related symptoms are more common and more severe in COPD patients than in other general medicine patients [2-4]. Excess reflux, as determined by pH-monitoring, has also been documented to be higher in COPD [5]. GERD has also been associated with more frequent COPD exacerbations [6,7]. The cause of this important association is unknown, but these data suggest not only that is GERD more common in COPD, but also that by increasing exacerbations, GERD may alter COPD presentation and course.

We hypothesized that COPD patients with GERD would have poorer QOL and more frequent exacerbations, but that the use of GERD-controlling medications, proton-pump inhibitors (PPIs) in particular, could modify these associations. To test this hypothesis, in the current observational study, combining cross-sectional and longitudinal data, we comprehensively evaluated clinical, physiologic and imaging differences between COPD patients with versus without a physician-based diagnosis of GERD. We also present an examination of how comorbid GERD impacts different measures of COPD-related health status and exacerbations.

## Methods

### Patient selection

The COPDGene Study (http://www.COPDGene.org/) is an ongoing NHLBI-funded multicenter study of the genetic epidemiology of smoking-related lung disease (Clinical Trials Registration # NCT00608764). A complete description of the protocol has already been published [8]. Briefly, inclusion criteria other than ability to give informed consent are: age 45-80 years; at least 10 pack-years cigarette smoking history; self-defined non-Hispanic white or African-American ancestry; and willingness to undergo study-related tests, including spirometry, CT scan of the chest and blood collection for biomarker and genetic analysis. For the current analysis, we selected from the full cohort of 10,300 enrolled subjects participants with COPD, both former or current smokers, who met criteria for GOLD stage 1 or greater (fixed airflow obstruction with a post-bronchodilator FEV_1_/FVC [forced vital capacity] ratio ≤0.7),. Additionally, all subjects had CT measurements of emphysema and airway abnormalities completed at the time of data analysis. Parenchymal analysis was performed using Slicer (http://www.Slicer.org); airway analysis was performed using VIDA Pulmonary Workstation 2 (http://www.vidadiagnostics.com). Lung areas with attenuation value of less than-950 Hounsfield Units (HU) on the inspiratory scans were considered emphysematous. CT-measurements of airway disease included mean bronchial wall thickness calculated as an average of six segmental values for each subject, wall area percent (100* wall area/total bronchial area) and the square root of the wall area of a theoretical airway of 10 mm luminal perimeter (Pi10) [8]. The research protocol was approved by the institutional review board at each participating institution (University of Michigan Health System Research Committee IRB approval HUM000014973, 07/16/2010), and all participants provided written informed consent.

### Data collection

Demographic data, smoking, and medical history were collected using self-administered questionnaires. Symptoms and self-reported acute exacerbation frequency were quantified using a modified version of the ATS Chronic Respiratory Disease Questionnaire (ATS-DLD-78) [9] with the question: “Have you had a flare-up of your chest trouble in the last 12 months?” If the answer was “No”, zero exacerbations were recorded, and when the answer was “Yes”, additional questions on the presence, severity, management and number of exacerbations followed. Dyspnea was assessed using the Modified Medical Research Council scale [10], health-related QOL with the St. George’s Respiratory Questionnaire (SGRQ) [11], Medical Outcomes Study Short Form (SF) 36 version 2, and the BODE index score was calculated for all patients [12]. Comorbidities were assessed based on subject self-report. At enrollment all participants agreed to be contacted on a regular basis and to provide information about development of new exacerbations in the interval since the initial visit, using the same initial questions. Participants were followed longitudinally via either an automated telephone or web-based system on an every 6-month basis. Subjects not reached through the automated system were contacted by a research coordinator for a phone-based interview. Both baseline history of exacerbations as well as exacerbations during longitudinal follow-up were dichotomized on “frequent or infrequent”, based on the currently accepted definition of ≥2 exacerbation per year [13].

### Physiologic testing

Participants underwent spirometry pre- and post- administration of short-acting bronchodilating medication (albuterol), using the EasyOne™ spirometry system (Zurich, Switzerland). Predictive values were obtained using NHANES III data [14]. Quality control was performed for all spirometry tests using both an automated system and manual review. Six minute walk distance was measured in standard fashion [15].

### Diagnosis of GERD and medication use

Ascertainment of physician-diagnosed GERD was based on self-report. The patient was presented with the question “Have you ever been told by a physician that you have…” and a list of different diseases, including GERD. Information on medications, including GERD-related prescriptions PPIs, antihistamines, antacids), was also collected.

### Statistical analysis

Clinical characteristics between patients with and without GERD were compared with t-tests or chi-square tests where appropriate. Multivariate linear regression was used to model SGRQ and MMRC scores. Given the skewed distribution of exacerbation frequency, we used a zero-inflated Poisson model. All models were adjusted for age, gender, current smoking history, BMI, FEV_1_% predicted and clinical center. In the final models, we also evaluated the influence of PPIs and of the GERD-PPIs interaction. Because GERD could be a marker for the presence of other serious diseases and conditions independently associated with worse COPD outcomes, we also conducted additional analyses to determine whether differences in the GERD population as compared to the entire population contributed to differences in outcomes. To better isolate the impact of GERD, we generated a covariate representing the propensity to be diagnosed with GERD, based on a logistic regression model. In this model, the response variable of GERD diagnosis was generated, using as predictors either the variables with association <0.10 in univariate analysis or those accepted in the literature as associated with GERD (age, gender, BMI, smoking history, presence of cardiovascular disease, osteoporosis and gastric ulcers) [16,17]. These estimated propensity scores were included as a covariate in fully-adjusted regression models for outcomes of interest. All statistical analyses were conducted using STATA v.12 statistical software (College Station, TX.). A p-value <0.05 was considered significant.

## Results

### GERD is associated with worse outcomes and greater use of respiratory medications in COPD

Data were available for 4,483 participants who met GOLD criteria for COPD, of any GOLD stage, and who had answered the question of whether they had been previously diagnosed by a physician as having GERD. Table 1 outlines patient demographics, clinical characteristics, spirometry, and disease impact (SGRQ, BODE, MMRC dyspnea score and exacerbation frequency), stratified by history of GERD. Physician-diagnosed GERD was reported by 29.1% of the subjects, more frequently among women (50.9% vs. 41.3%, p < 0.0001) and older individuals (63.9 vs. 62.7 years of age, p < 0.0001). Statistical differences of no clinical significance were found in severity of airflow obstruction among GERD subjects (56.3% vs. 57.9%, p = 0.02) as well as those with higher BMI (28.8 kg/m^2^ vs. 27.5 kg/m^2^, p <0.0001); the distribution by GOLD stages was also similar. Although those with GERD reported greater tobacco consumption (53.4 vs. 50.0 pack-years, p = 0.003), they were less frequently current smokers (34.3% vs. 47.1%, p < 0.0001). Compared to patients with COPD without GERD, a greater frequency of subjects reporting coexistent GERD also fulfilled the diagnostic criteria for chronic bronchitis (29.0% vs. 24.7%, p = 0.002). Subjects with coexistent GERD were more frequently prescribed all classes of COPD-related medications, including short-acting medications (61.0% vs. 48.5%, p < 0.0001) and controller medications (e.g. combined steroid and long-acting beta-agonist 41.7% vs. 33.6%, p < 0.0001; long-acting anti-muscarinics 40.2% vs. 30.8%, p < 0.0001).

**Table 1:** Demographics, spirometry, and clinical characteristics of COPD subjects stratified by history of GERD*

	**GERD (*n*** **= 1,307)**	**No GERD (*n *** **=3,176)**	**Total (*n *** **=4,483)**	**P-value**
Age (years) [mean (s.d.)]	63.9 (8.2)	62.7 (8.8)	63.1 (8.6)	<0.0001
Gender (% female)	50.9	41.3	44.1	<0.0001
FEV1% predicted [mean (s.d.)]	56.3 (21.4)	57.9 (23.3)	57.4 (22.8)	0.02
Spirometry Gold Stage (%)				
Stage 1-2	60.1	60.7	60.6	
Stage 3-4	39.9	39.3	39.4	0.67
Body mass index (kg/m^2^) [mean (s.d.)]	28.8 (6.1)	27.5 (6.1)	27.9 (6.1)	<0.0001
Current smoking (% of each group)	34.3	47.1	43.3	<0.0001
Pack years [mean (s.d.)]	53.4 (27.6)	50.8 (27.0)	51.6 (27.2)	0.003
Currently works (% patients)	24.4	28.0	26.9	0.01
Education beyond high school (% patients)	60.9	59.7	60.1	0.45
Chronic bronchitis (% patients)	29.0	24.7	25.9	0.002
Short-acting beta-agonists (% of patients)	61.0	48.5	52.1	<0.0001
Long-acting beta-agonists [LABA] (% of patients)	9.1	6.5	7.3	0.011
Inhaled corticosteroids [ICS] (% of patients)	13.9	9.3	10.6	<0.0001
Combination ICS/LABA (% of patients)	41.7	33.6	36.0	<0.0001
Long acting anti-muscarinic (% of patients)	40.2	30.8	33.5	<0.0001
Any GERD therapy (% of patients)	52.8	8.2	21.2	<0.0001
Proton Pump Inhibitors (% of patients)	46.8	6.5	18.2	<0.0001

*Means and standard deviations of the mean or percentages are presented; p-values represent *t*-test or chi-square comparison between groups.

### GERD is associated with worse QOL and greater co-morbidity in COPD

Significant differences were also noted in the impact of GERD on QOL measures and exacerbations (Table 2). Those with GERD had a shorter six minute walk distance (1,192 vs. 1,250 feet, p < 0.0001); higher BODE index (2.7 vs. 2.4, p = 0.001), more severe MMRC dyspnea scores (2.2 vs. 1.8, p < 0.0001); higher total SGRQ score (41.8 points vs. 34.9 points, p < 0.0001); lower SF36 PCS score (38.2 vs. 41.4 points, p < 0.0001), and more frequent exacerbations in the year prior to enrollment (0.9 vs. 0.6, p < 0.0001). Importantly, those with GERD more frequently reported ≥2 exacerbations in the prior year (22.5% vs. 12.9% of those without GERD, p < 0.001).

**Table 2:** Comorbidities, disease impact and health-related quality-of-life stratified by history of GERD*

	**GERD (*n*** **= 1,307)**	**No GERD (*n *** **=3,176)**	**Total (n = 4,483)**	**P-value**
**Comorbidities**				
Cardiovascular disease**	21.3	14.0	16.1	<0.0001
Congestive heart failure	5.7	4.1	4.6	0.01
Hypertension	55.7	45.2	48.3	<0.0001
Diabetes mellitus	13.5	11.7	12.3	0.08
Peripheral vascular disease	4.7	2.5	3.2	0.0001
Osteoporosis/Compression fractures	25.0	13.4	16.8	<0.0001
Any arthritis	28.6	15.8	19.5	<0.0001
Sleep apnea	22.2	13.5	16.1	<0.0001
2 or more modifiable risk factors***	60.5	50.5	53.4	<0.0001
**Disease impact and health-related quality-of-life**				
Six minute walk distance (feet) [mean (s.d.)]	1,192 (392)	1,250 (413)	1,233 (408)	<0.0001
BODE [mean (s.d.)]	2.7 (2.0)	2.4 (2.1)	2.5 (2.1)	0.001
MMRC dyspnea score [mean (s.d.)]	2.2 (1.4)	1.8 (1.5)	1.9 (1.5)	<0.0001
SGRQ total score [mean (s.d.)]	41.8 (22.3)	34.9 (22.9)	36.9 (22.9)	<0.0001
SF-36 PCS	38.2 (10.4)	41.4 (11.2)	40.4 (11.0)	<0.0001
SF-36 MCS	48.0 (13.2)	49.1 (12.3)	48.8 (12.6)	0.07
Exacerbation in the year prior to enrollment [mean (s.d.)]	0.9 (1.3)	0.6 (1.1)	0.7 (1.2)	<0.0001
Hospitalized exacerbations in the year prior to enrollment (%)	22.0	18.6	19.5	0.008
Frequent exacerbator phenotype [>/=2 exacerbations per annum] (%)	22.5	12.9	15.7	<0.0001
Exacerbations per year during follow-up [mean (s.d.)]	0.9 (1.7)	0.6 (1.6)	0.7 (1.6)	<0.0001
Frequent exacerbator phenotype during follow-up [>/=2 exacerbations per annum] (%)	17.9	14.7	16.9	<0.0001

*Percentages are presented; p-values represent chi-square comparison between groups.

**As a combination of Myocardial Infarction, Coronary Artery Disease, and Stroke/TIA.

***Modifiable cardiovascular disease risk factors include hypertension, hyperlipidemia, current smoking, and BMI > 30.

COPD subjects with GERD also had significantly increased frequency of comorbidities (Table 2). We found significantly increased frequency of myocardial infarction (10.9% vs. 6.4%, p < 0.0001), coronary artery disease (11.9% vs. 7.6%, p < 0.0001), angina (8.6% vs. 4.0%, p < 0.0001), as well as modifiable cardiovascular risk factors (two or more of the following: hypertension, hyperlipidemia, obesity and smoking, 60.5% vs. 50.5%, p < 0.0001), and sleep apnea (22.2% vs. 13.5%, p < 0.0001). By contrast, we found no differences in the reported frequency of diabetes (13.5% vs. 11.7%, p = 0.22).

### GERD is independently associated with worse QOL, more frequent exacerbation of COPD & clinical but not radiographic markers of microaspiration

After identifying the imbalance between GERD and no-GERD patients in the distribution of demographics and comorbid conditions, we generated a propensity score for GERD diagnosis, as described in the Methods section. This propensity score was well-balanced with respect to the distribution of important covariates. Next, in separate multivariate models, each controlling for age, gender, BMI, current smoking and severity of airflow obstruction, we examined the effect of GERD on health-related QOL and exacerbation frequency in COPD. The three models tested the independent effect of GERD (model 1); of GERD plus propensity to be diagnosed with GERD (model 2); and with the addition of use of PPIs plus GERD-PPI use interaction (model 3) (Table 3).

**Table 3:** Multivariate models of the effect of GERD on different COPD outcomes among participants in COPDGene (n = 4,483)*

	**Model 1 GERD diagnosis Regression coefficient or OR (95% CI)**	**Model 2 Additionally adjusted for propensity to be diagnosed with GERD Regression coefficient or OR (95% CI)**	**Model 3 Additionally adjusted for PPIs use and GERD-PPIs Interaction Regression coefficient or OR (95% CI)**
Outcome: SGRQ score symptoms			
GERD	7.40 (5.93, 8.87)	2.48 (1.01, 3.95)	3.40 (1.56, 5.22)
PPI use			4.19 (1.09, 7.28)
GERD*PPIs interaction (p-value)			0.006
Outcome: SGRQ score activity			
GERD	6.35 (4.83, 7.81)	3.00 (1.46, 4.55)	3.82 (1.90, 5.75)
PPI use			3.63 (0.37, 6.88)
GERD*PPIs interaction (p-value)			0.021
Outcome: SGRQ score impact			
GERD	5.48 (4.27, 6.69)	2.34 (1.10, 3.58)	3.09 (1.55, 4.64)
PPI use			3.75 (1.14, 6.36)
GERD*PPIs Interaction (p-value)			0.005
Outcome: SGRQ total score			
GERD	6.05 (4.85, 7.26)	2.58 (1.36, 3.80)	3.38 (1.86, 4.90)
PPI use			3.77 (1.19, 6.34)
GERD*PPIs interaction (p-value)			0.003
Outcome: Count of exacerbations in the year prior to enrollment**			
GERD	1.32 (1.21, 1.44)	1.16 (1.06, 1.27)	1.17 (1.05, 1.31)
PPI use			1.20 (1.00, 1.44)
GERD*PPIs interaction			NS
Outcome: Frequent exacerbator at enrollment			
GERD	1.88 (1.57, 2.24)	1.41 (1.17, 1.71)	1.40 (1.10, 1.77)
PPI use			1.60 (1.09, 2.35)
GERD*PPIs interaction			NS
Outcome: Frequent exacerbator at follow-up			
GERD	1.81 (1.52, 2.16)	1.50 (1.24, 1.82)	1.40 (1.10, 1.79)
PPI Use			1.56 (1.06, 2.30)
GERD*PPIs interaction			NS

*All models additionally adjusted for age, gender, BMI, current smoking, FEV1% predicted and clinical center.

**Zero-inflated Poisson model.

These analyses demonstrate that the presence of GERD in COPD is associated with worse QOL, with an associated 3.4 point (95% CI 1.8, 4.9) higher SGRQ total score, which is within the accepted minimal important difference for this measure of health status [18].

Using data on enrollment, GERD in COPD is also associated with higher frequency of exacerbations in the previous year (1.17 fold increase in frequency; 95% CI 1.05, 1.31; p = 0.005) and more risk of being a “frequent exacerbator” (OR 1.40; 95% CI 1.10, 1.77; p = 0.006). Additional analysis after a mean follow-up of two years showed a sustained association between GERD and exacerbation frequency fulfilling the definition of frequent exacerbation (OR 1.40; 95% CI 1.10, 1.79; p = 0.006).

All models (Table 3, third column) include an analysis of the effect of PPIs. They uniformly demonstrate that PPI use does not change the associations between GERD and exacerbations and are actually associated with poor outcomes. Further analysis of the interaction of GERD by PPI use demonstrated significance only when modeling QOL, but not for exacerbations. In additional sensitivity analyses we included in the propensity score and all multivariate models the imbalance in the use of respiratory medications, and there was not change in the strength or the direction of the associations.

To explore hypothetical mechanistic explanations for GERD-COPD outcome relationships, we also used our data to examine two additional associations. First, we looked for differences in indirect markers of recurrent aspiration or microaspiration, and found all to be more frequent among those with GERD: reported pneumonia (58.1% vs. 43.9%, p < 0.0001); recurrent wheezing (69.8% vs. 55.5%, p < 0.0001); and physician-diagnosed asthma (28.8% vs. 20.0%, p < 0.0001). Second, we searched for radiologic changes that might accompany GERD. This analysis disclosed no differences between COPD patients with versus without GERD in severity of emphysema, or in any of the measures of airway thickness (Table 4).

**Table 4:** Aspiration-related symptoms, and radiologic measurements stratified by history of GERD*

	**GERD (*n*** **= 1,307)**	**No GERD (*n *** **=3,176)**	**Total (*n *** **=4,483)**	**P-value**
History of Pneumonia (%)	58.1	43.9	48.1	<0.0001
Asthma (%)	28.8	20.0	22.6	<0.0001
Have a cough (%)	47.4	41.7	43.3	0.0004
Have wheezing (%)	69.8	55.5	59.7	<0.0001
CT calculated TLC, liters [mean (s.d.)]	5.9 (1.4)	6.0 (1.4)	6.0 (1.4)	0.003
Emphysema [mean (s.d.)]	11.9 (12.1)	11.3 (12.1)	11.5 (12.1)	0.20
Emphysema >20% (%)	23.9	22.9	23.2	0.12
% Gas trapping [mean (s.d.)]	35.9 (20.0)	35.4 (21.2)	35.5 (20.1)	0.40
Wall thickness in mm [mean (s.d.)]	62.4 (3.2)	62.4 (3.2)	62.4 (3.2)	0.90
Pi10	3.7 (0.1)	3.7 (0.1)	3.7 (0.1)	0.30

*Means and standard deviations of the mean or percentages are presented; p-values represent *t*-test or chi-square comparison between groups.

## Discussion

The analysis of a large cross-sectional multicenter study of patients with a wide range of COPD severities provides evidence of a significant association between the presence of GERD and both more symptomatic disease and poorer health status. We found that GERD is frequently found among COPD patients, and that COPD patients with coexistent GERD experience worse QOL (SGRQ), greater dyspnea (MMRC), and more frequent exacerbations during follow-up. These associations were maintained in multivariate analysis, independent of the effect of age, gender, severity of airflow obstruction, current smoking history and BMI. Importantly, these associations persisted after adjusting both for therapeutic PPI use and for the imbalance of other covariates associated with GERD diagnosis. Based on additional analysis of the GERD-PPi use interaction (model 3), we also discovered that PPIs use itself could be associated with more frequent exacerbations, but also with improved health-related QOL, as measured by SGRQ.

The 29.1% prevalence of physician-diagnosed GERD in our population is higher than the 16.5% reported by Bor [3], which used different disease-specific questionnaires; it is lower than reports based on continuous esophageal pH monitoring [5], but within the same range of other reports based on frequency of symptoms [4] and self-report data [19] in different clinical settings and in different countries [20]. Our finding of a similar degree of airflow obstruction, measured by FEV_1_% predicted, among COPD patients regardless of GERD diagnosis agrees with and greatly extends data presented in smaller series [2,5]. Interestingly, even with similar spirometry values, our participants with GERD had more intense bronchodilator and anti-inflammatory treatment, most probably in response to more frequent symptoms and exacerbations. The slightly greater frequency of GERD in women with COPD agrees with general population-based studies [21] and recent inquires of healthcare utilization in the US [22]. Whether this greater frequency of GERD in women with COPD is casually responsible of the established finding from multiple studies that women with COPD also suffer more frequent exacerbations [23,24], is an important topic for future investigation in this and other large longitudinal studies.

Although anticipated, our finding that multiple comorbidities were significantly more frequent in COPD patients with GERD is significant given our standardized physiological and radiographic characterization of COPD phenotypes and the potential to look for genetic association between COPD and its comorbidities in future analysis of this cohort. Those GERD-associated comorbidities in COPD included cardiovascular diseases, both as manifestations of established disease (including myocardial infarction, coronary artery disease, stroke/TIA), as well as cardiovascular risk factors (including obesity, current tobacco use, hypertension, and hyperlipidemia). Clustering of these comorbidities could reflect shared risk factors (obesity and diet) or a common inflammatory process, ultimately affecting the progression of COPD [25]. We also tested if differences in our findings are explained by other factors (e.g. sleep apnea), and found that the GERD-COPD outcomes relations are maintained in the presence or absence of sleep apnea (Additional file 1: Table S1). The higher frequency of poor outcomes among participants with GERD in our longitudinal study supports the clinical suspicion that GERD could be an indicator of more frequent comorbidities.

We attempted to overcome two major limitations of previous observational studies examining the association between GERD and COPD. The first is the already described finding of clustering of comorbidities and poorer baseline functional status of those with GERD, which complicates isolating the effect of GERD alone. Hence, we performed a propensity analysis to identify additional factors associated with a diagnosis of GERD, and showed that the association between GERD and worse COPD outcomes were maintained. A second novel feature of our study is that we adjusted for PPI use and GERD-PPI use interactions. The independent association of PPI use with exacerbations and QOL may be due to confounding-by-indication, such that such individuals were deemed “sicker” by their physicians and therefore more likely to receive PPI therapy for their GERD. Our results overall, however, support a relationship between GERD and poor COPD outcomes, even appropriately controlling for the effect of medications.

It is noteworthy that our COPD patients with GERD more frequently displayed clinical characteristics of the chronic bronchitis predominant COPD phenotype recently described using data from this same cohort [26]. Sputum production is gaining more acceptance as a predictor of increased risk for frequent exacerbations and for response to specific therapies [27]. It is possible that the presence of GERD increases bronchial inflammation in COPD patients; however, we did not find a difference in airway disease measures assessed via imaging between those with GERD versus without GERD. Others have reported an association between an inflammatory marker, exhaled breath condensate pH, and GERD symptoms in COPD patients [4], but further studies are needed to understand fully this potential relationship and its potential treatment implications.

The finding that GERD in COPD patients was associated with poorer QOL and more severe dyspnea agrees and significantly extends previous descriptions. In a study of 86 patients with COPD who answered a questionnaire to identify GERD symptoms, Rascon-Aguilar et al. [28] showed univariate differences in SF-36 QOL scores related to the presence GERD symptoms, of the same magnitude we detected. We extend those findings to show that the association between reflux and poorer health-related QOL (measured with the SGRQ questionnaire) persists in multivariate analyses controlled for the effects of age, gender, pulmonary function, current smoking, and BMI. Importantly, the statistically significant effect in our data persists after controlling for medication. Although GERD therapy has been reported to improve QOL among the general population [29], whether the same occurs among COPD patients is unclear.

The current finding that GERD is significantly associated with an exacerbation history also agrees with Hurst et al. [7], who found an association with similar strength, but who did not control for the effect of PPIs use. The consistent results from different research designs in different populations [7,30] point towards a significant relationship between GERD and COPD exacerbations, and provide a robust body of evidence to give strong consideration to this factor as a potential disease-modifying intervention. At least one small study has tested the concept of using PPIs in COPD patients with no reflux symptoms [31], and found a lower number of exacerbations after treatment with lansoprazole. However, our data generate additional questions about the potential use of PPIs to modify some COPD outcomes, as the association differed in our study depending on whether the outcome is QOL or exacerbation frequency. We have highlighted to the possibility of confounding-by-indication in the use of PPIs, which needs to be addressed in future studies. An intriguing possibility, which will require additional investigation, is that control of acid aspiration improves QOL, but that the association of GERD with increased exacerbation frequency relates less closely to acid aspiration than to changes in the community structure of the lung microbiome [32], as suggested by findings of higher frequency of pneumonia among PPIs users in the general population [33,34].

Our study has several limitations. GERD diagnosis was based on self-report of a physician diagnosis, not on pH probe measures or specific questionnaires, which is potentially subject to recall bias. Furthermore, even physician diagnosis of GERD may not correlate with pH probe testing. Additionally, the design of COPDGene is not population-based, although it is inclusive of all levels of COPD severity and includes subjects recruited across the country at a wide variety of clinical centers. Whether the associations between the outcomes of interest and GERD therapy are restricted to PPIs, and not to antihistamines or other anti-GERD medications, is difficult to answer; only 3.3% of our participants were exposed to antihistamines, limiting the power for specific analysis. The current study was not designed to ascertain compliance or persistence on medications, and inquired about PPIs at inclusion, not persistence on therapy, a factor potentially affecting results in longitudinal studies. The strengths of our study include the extensive clinical and epidemiological characterization of the participants; the number and heterogeneity of subjects with versus without GERD included in the analysis; and our efforts to control for confounders common in observational studies, such as clustering of comorbidities and ignoring the effect of concurrent treatments.

We conclude that presence of GERD in COPD is associated with increased symptoms, poorer health-related QOL and increased frequency of exacerbations, even after adjusting for a self-reported use of GERD-related medication. Because a history of GERD identifies COPD patients at risk for poorer clinical outcomes, our results imply that identification and management of GERD should be considered part of optimal clinical management of COPD. Further research in the area is neccesary. Clinical trials to identify and control GERD to improve COPD outcomes should be considered.

## Abbreviations

COPD: Chronic obstructive pulmonary disease; GERD: Gastroesophageal reflux disease; PPIs: Proton-pump inhibitors; BMI: Body mass index; MMRC: Modified Medical Research Council dyspnea scale; QOL: Quality of life; SGRQ: Saint George’s respiratory questionnaire; SF36: Medical outcomes study short form health survey, 36-item; NHLBI: National Heart, Lung and Blood Institute; CT: Computed tomography; FEV: Forced expiratory volume in the first second; FVC: Forced vital capacity; HU: Hounsfield units; Pi10: Theoretical airway of 10 mm luminal perimeter; ATS: American Thoracic Society; NHANES: National health and nutrition examination survey; TIA: Transient ischemic attack.

## Competing interests

The authors have reported the following conflicts of interest: Dr. Crapo received travel accommodations from AstraZeneca. Dr. F.J. Martinez has been a member of advisory boards for Actellion, Ikaria, Merck, Pearl, Pfizer, Jannsen, GlaxoSmithKline plc, Schering Plough, Novartis AG, Nycomed, Genzyme, Forest/Almirall, MedImmune, AstraZeneca, Potomac, Bayer, Elan, Talecris, and Roche. He has been on the speaker’s bureau for Forest/Almirall, Nycomed, Bayer, Boehringer Ingelheim GmbH, GlaxoSmithKline plc, France Foundation, MedEd, NACE, and AstraZeneca. He has also been a member of steering committees for studies supported by Altana/Nycomed, GlaxoSmithKline, Gilead, Actelion, Johnson/Johnson, Mpex, UCB, and the National Institutes of Health. He has been an investigator in trials supported by Boehringer Ingelheim and Actelion. Dr. Han participated in advisory boards for Boehringer Ingelheim GmbH, Pfizer, GlaxoSmithKline plc, Genentech, Novartis, Forest and Medimmune; participated on speaker’s bureaus for Boehringer Ingelheim GmbH, Pfizer, GlaxoSmithKline plc, Grifols Therapeutics, Forest and the National Association for Continuing Education, and WebMD; has consulted for Novartis and United Biosource Corporation; and has received royalties from UpToDate and ePocrates. Drs. C.H. Martinez, Okajima, Murray, Silverman, Lee, Regan, Curtis and Hatabu, have reported that no potential conflicts of interest exist with any companies/organizations whose products or services may be discussed in this article.

## Authors’ contributions

CHM, MKH, SH, FJM, JLC, GRW, EAR contributed to the design, data collection, data analysis and interpretation and drafted the manuscript. YO, EKS, JHL, JDC, HH contributed to the design, data analysis and interpretation and drafted the manuscript. All authors have given final approval of the version to be published.

## Supplementary Material

Additional file 1: Table S1 Demographics, spirometry, and clinical characteristics of COPD subjects stratified by history of GERD and Sleep Apnea. Click here for file

## References

[B1] El-Serag HB Sonnenberg A Comorbid occurrence of laryngeal or pulmonary disease with esophagitis in United States military veterans Gastroenterology 1997 113 3 755 760 10.1016/S0016-5085(97)70168-9 9287965

[B2] Mokhlesi B Morris AL Huang CF Curcio AJ Barrett TA Kamp DW Increased prevalence of gastroesophageal reflux symptoms in patients with COPD Chest 2001 119 4 1043 1048 10.1378/chest.119.4.1043 11296167

[B3] Bor S Kitapcioglu G Solak ZA Ertilav M Erdinc M Prevalence of gastroesophageal reflux disease in patients with asthma and chronic obstructive pulmonary disease J Gastroenterol Hepatol 2010 25 2 309 313 10.1111/j.1440-1746.2009.06035.x 19817951

[B4] Terada K Muro S Sato S Ohara T Haruna A Marumo S Kinose D Ogawa E Hoshino Y Niimi A Terada T Mishima M Impact of gastro-oesophageal reflux disease symptoms on COPD exacerbation Thorax 2008 63 11 951 955 10.1136/thx.2007.092858 18535116

[B5] Casanova C Baudet JS del Valle VM Martin JM Aguirre-Jaime A de Torres JP Celli BR Increased gastro-oesophageal reflux disease in patients with severe COPD Eur Respir J 2004 23 6 841 845 15218995 10.1183/09031936.04.00107004

[B6] Rascon-Aguilar IE Pamer M Wludyka P Cury J Coultas D Lambiase LR Nahman NS Vega KJ Role of gastroesophageal reflux symptoms in exacerbations of COPD Chest 2006 130 4 1096 1101 10.1378/chest.130.4.1096 17035443

[B7] Hurst JR Vestbo J Anzueto A Locantore N Mullerova H Tal-Singer R Miller B Lomas DA Agusti A Macnee W Calverley P Rennard S Wouters EF Wedzicha JA Evaluation of COPD Longitudinally to Identify Predictive Surrogate Endpoints (ECLIPSE) Investigators Susceptibility to exacerbation in chronic obstructive pulmonary disease N Engl J Med 2010 363 12 1128 1138 10.1056/NEJMoa0909883 20843247

[B8] Regan EA Hokanson JE Murphy JR Make B Lynch DA Beaty TH Curran-Everett D Silverman EK Crapo JD Genetic epidemiology of COPD (COPDGene) study design COPD 2010 7 1 32 43 10.3109/15412550903499522 20214461 PMC2924193

[B9] Ferris BG Epidemiology Standardization Project (American Thoracic Society) Am Rev Respir Dis 1978 118 6 Pt 2 1 120 742764

[B10] Bestall JC Paul EA Garrod R Garnham R Jones PW Wedzicha JA Usefulness of the Medical Research Council (MRC) dyspnoea scale as a measure of disability in patients with chronic obstructive pulmonary disease Thorax 1999 54 7 581 586 10.1136/thx.54.7.581 10377201 PMC1745516

[B11] Jones PW Quirk FH Baveystock CM The St George’s respiratory questionnaire Respir Med 1991 85 Suppl B 25 31 discussion 33–27 1759018 10.1016/s0954-6111(06)80166-6

[B12] Celli BR Cote CG Marin JM Casanova C de Oca Montes M Mendez RA Pinto Plata V Cabral HJ The body-mass index, airflow obstruction, dyspnea, and exercise capacity index in chronic obstructive pulmonary disease N Engl J Med 2004 350 10 1005 1012 10.1056/NEJMoa021322 14999112

[B13] Wedzicha JA Brill SE Allinson JP Donaldson GC Mechanisms and impact of the frequent exacerbator phenotype in chronic obstructive pulmonary disease BMC Med 2013 11 181 10.1186/1741-7015-11-181 23945277 PMC3750926

[B14] Hankinson JL Odencrantz JR Fedan KB Spirometric reference values from a sample of the general U.S. population Am J Respir Crit Care Med 1999 159 1 179 187 10.1164/ajrccm.159.1.9712108 9872837

[B15] Anonymous ATS statement: guidelines for the six-minute walk test Am J Respir Crit Care Med 2002 166 1 111 117 12091180 10.1164/ajrccm.166.1.at1102

[B16] Adamina M Guller U Weber WP Oertli D Propensity scores and the surgeon Br J Surg 2006 93 4 389 394 10.1002/bjs.5265 16400708

[B17] Rubin DB The design versus the analysis of observational studies for causal effects: parallels with the design of randomized trials Stat Med 2007 26 1 20 36 10.1002/sim.2739 17072897

[B18] Gross NJ Chronic obstructive pulmonary disease outcome measurements: what’s important? What’s useful? Proc Am Thorac Soc 2005 2 4 267 271 discussion 290-261 10.1513/pats.200504-036SR 16267347

[B19] Agusti A Calverley PM Celli B Coxson HO Edwards LD Lomas DA MacNee W Miller BE Rennard S Silverman EK Tal-Singer R Wouters E Yates JC Vestbo J Evaluation of COPD Longitudinally to Identify Predictive Surrogate Endpoints (ECLIPSE) investigators Characterisation of COPD heterogeneity in the ECLIPSE cohort Respir Res 2010 11 12220831787

[B20] Kim J Lee JH Kim Y Kim K Oh YM Yoo KH Rhee CK Yoon HK Kim YS Park YB Lee SW Lee SD Association between chronic obstructive pulmonary disease and gastroesophageal reflux disease: a national cross-sectional cohort study BMC Pulm Med 2013 13 1 51 10.1186/1471-2466-13-51 23927016 PMC3750392

[B21] Zagari RM Fuccio L Wallander MA Johansson S Fiocca R Casanova S Farahmand BY Winchester CC Roda E Bazzoli F Gastro-oesophageal reflux symptoms, oesophagitis and Barrett’s oesophagus in the general population: the Loiano-Monghidoro study Gut 2008 57 10 1354 1359 10.1136/gut.2007.145177 18424568

[B22] Friedenberg FK Hanlon A Vanar V Nehemia D Mekapati J Nelson DB Richter JE Trends in gastroesophageal reflux disease as measured by the National Ambulatory Medical Care Survey Dig Dis Sci 2010 55 7 1911 1917 10.1007/s10620-009-1004-0 19830561

[B23] Tashkin D Celli B Kesten S Lystig T Decramer M Effect of tiotropium in men and women with COPD: results of the 4-year UPLIFT trial Respir Med 2010 104 10 1495 1504 10.1016/j.rmed.2010.03.033 20418083

[B24] Celli B Vestbo J Jenkins CR Jones PW Ferguson GT Calverley PM Yates JC Anderson JA Willits LR Wise RA Sex differences in mortality and clinical expressions of patients with chronic obstructive pulmonary disease. The TORCH experience Am J Respir Crit Care Med 2011 183 3 317 322 10.1164/rccm.201004-0665OC 20813884

[B25] Martinez CH Han MK Contribution of the environment and comorbidities to chronic obstructive pulmonary disease phenotypes Med Clin North Am 2012 96 4 713 727 10.1016/j.mcna.2012.02.007 22793940 PMC4629222

[B26] Kim V Han MK Vance GB Make BJ Newell JD Hokanson JE Hersh CP Stinson D Silverman EK Criner GJ The Chronic Bronchitic Phenotype of COPD: an analysis of the COPDGene Study Chest 2011 140 3 626 633 10.1378/chest.10-2948 21474571 PMC3168856

[B27] Rennard SI Calverley PM Goehring UM Bredenbroker D Martinez FJ Reduction of exacerbations by the PDE4 inhibitor roflumilast-the importance of defining different subsets of patients with COPD Respir Res 2011 12 18 10.1186/1465-9921-12-18 21272339 PMC3040135

[B28] Rascon-Aguilar IE Pamer M Wludyka P Cury J Vega KJ Poorly treated or unrecognized GERD reduces quality of life in patients with COPD Dig Dis Sci 2011 56 7 1976 1980 10.1007/s10620-010-1542-5 21221789

[B29] Havelund T Lind T Wiklund I Glise H Hernqvist H Lauritsen K Lundell L Pedersen SA Carlsson R Junghard O Stubberöd A Anker-Hansen O Quality of life in patients with heartburn but without esophagitis: effects of treatment with omeprazole Am J Gastroenterol 1999 94 7 1782 1789 10.1111/j.1572-0241.1999.01206.x 10406235

[B30] Han MK Kazerooni EA Lynch DA Liu LX Murray S Curtis JL Criner GJ Kim V Bowler RP Hanania NA Anzueto AR Make BJ Hokanson JE Crapo JD Silverman EK Martinez FJ Washko GR COPDGene Investigators Chronic obstructive pulmonary disease exacerbations in the COPDGene study: associated radiologic phenotypes Radiology 2011 261 1 274 10.1148/radiol.11110173 21788524 PMC3184233

[B31] Sasaki T Nakayama K Yasuda H Yoshida M Asamura T Ohrui T Arai H Araya J Kuwano K Yamaya M A randomized, single-blind study of lansoprazole for the prevention of exacerbations of chronic obstructive pulmonary disease in older patients J Am Geriatr Soc 2009 57 8 1453 1457 10.1111/j.1532-5415.2009.02349.x 19515110

[B32] Han MK Huang YJ Lipuma JJ Boushey HA Boucher RC Cookson WO Curtis JL Erb-Downward J Lynch SV Sethi S Toews GB Young VB Wolfgang MC Huffnagle GB Martinez FJ Significance of the microbiome in obstructive lung disease Thorax 2012 67 5 456 463 10.1136/thoraxjnl-2011-201183 22318161 PMC3578398

[B33] Gulmez SE Holm A Frederiksen H Jensen TG Pedersen C Hallas J Use of proton pump inhibitors and the risk of community-acquired pneumonia: a population-based case–control study Arch Intern Med 2007 167 9 950 955 10.1001/archinte.167.9.950 17502537

[B34] Eurich DT Sadowski CA Simpson SH Marrie TJ Majumdar SR Recurrent community-acquired pneumonia in patients starting acid-suppressing drugs Am J Med 2010 123 1 47 53 10.1016/j.amjmed.2009.05.032 20102991

