# Supplementary material for: Impact of self-reported Gastroesophageal reflux disease in subjects from COPDGene cohort
Source: Respir Res. 2014 Jun 3;15(1):62. doi: 10.1186/1465-9921-15-62 (PMC4049804; doi:10.1186/1465-9921-15-62)
Supplement: Additional file 1: Table S1 — Demographics, spirometry, and clinical characteristics of COPD subjects stratified by history of GERD and Sleep Apnea. [file 1465-9921-15-62-S1.docx]

**Additional file 1: Table S1. Demographics, spirometry, and clinical characteristics of COPD subjects stratified by history of GERD and Sleep Apnea.**

|  | Sleep Apnea present | | Sleep apnea absent | |
| --- | --- | --- | --- | --- |
|  | GERD | No GERD | GERD | No GERD |
| Demographic and clinical characteristics | | | | |
| Age (years) [mean (s.d.)] | 63.3 (8.1) | 63.9 (8.5) | 64.2 (8.2) | 62.8 (8.9) |
| Gender (% female) | 42.1 | 27.0 | 53.0 | 44.0 |
| FEV1% predicted [mean (s.d.)] | 56.0 (19.5) | 55.5 (21.5) | 56.4 (22.3) | 58.5 (23.6) |
| Spirometry Gold Stage (%) |  |  |  |  |
| Stage 1-2 | 61.7 | 57.0 | 59.6 | 61.7 |
| Stage 3-4 | 38.3 | 43.0 | 40.5 | 38.3 |
| Body mass index (kg/m^2^) [mean (s.d.)] | 32.1 (6.8) | 32.0 (7.1) | 27.6 (5.5) | 26.6 (5.4) |
| Current smoking (% of each group) | 29.3 | 36.3 | 35.4 | 48.5 |
| Pack years [mean (s.d.)] | 56.4 (28.7) | 54.7 (29.4) | 52.8 (27.4) | 49.9 (26.5) |
| Currently works (% patients) | 20.7 | 24.4 | 25.5 | 28.6 |
| Education beyond high school (% patients) | 39.0 | 38.1 | 59.4 | 59.1 |
| Chronic bronchitis (% patients) | 34.8 | 33.5 | 25.2 | 22.2 |
| Short-acting beta-agonists (% of patients) | 43.3 | 35.2 | 31.2 | 21.6 |
| Long-acting beta-agonists [LABA] (% of patients) | 39.7 | 40.5 | 35.6 | 25.8 |
| Inhaled corticosteroids [ICS] (% of patients) | 47.1 | 39.3 | 35.7 | 25.5 |
| Combination ICS/LABA (% of patients) | 45.0 | 36.5 | 30.7 | 24.1 |
| Long acting anti-muscarinic (% of patients) | 40.5 | 40.0 | 33.0 | 23.3 |
| Any GERD therapy (% of patients) | 53.1 | 12.1 | 52.5 | 7.2 |
| Proton Pump Inhibitors (% of patients) | 49.0 | 9.8 | 46.2 | 5.6 |
| Disease impact | | | | |
| Six minute walk distance (feet) [mean (s.d.)] | 1,110 (407) | 1,163 (410) | 1,219 (384) | 1,270 (412) |
| BODE [mean (s.d.)] | 2.9 (1.9) | 2.7 (2.0) | 2.6 (2.1) | 2.3 (2.2) |
| MMRC dyspnea score [mean (s.d.)] | 2.5 (1.3) | 2.2 (1.4) | 2.0 (1.4) | 1.7 (1.5) |
| SGRQ total score [mean (s.d.)] | 47.6 (21.0) | 42.4 (21.8) | 38.8 (21.9) | 32.4 (22.6) |
| Exacerbation in the year prior to enrollment [mean (s.d.)] | 1.0 (1.4) | 0.8 (1.3) | 0.8 (1.3) | 0.5 (1.1) |
| Frequent exacerbator phenotype [>/=2 exacerbations per annum] (%) | 27.6 | 17.2 | 20.1 | 11.3 |
| Frequent exacerbator phenotype during follow-up [>/=2 exacerbations per annum] (%) | 26.2 | 17.4 | 20.9 | 13.2 |
